# Taxonomy of *Hyphodermella*: a case study to show that simple phylogenies cannot always accurately place species in appropriate genera

**DOI:** 10.1186/s43008-023-00116-7

**Published:** 2023-06-06

**Authors:** Shan Shen, Shi-Liang Liu, Li-Wei Zhou

**Affiliations:** 1 State Key Laboratory of Mycology, Institute of Microbiology, Chinese Academy of Sciences, Beijing, 100101 People’s Republic of China; 2 University of Chinese Academy of Sciences, Beijing, 100049 People’s Republic of China

**Keywords:** Wood-inhabiting fungi, *Basidiomycota*, *Phanerochaetaceae*, *Pseudohyphodermella*, *Roseograndinia*, Five new taxa

## Abstract

The genus is a special and crucial taxonomic rank compared with others above the species level, because a species has to be placed in a certain genus instead of any other higher ranks. With more and more new species being described, the placements of their generic position are sometimes incorrect due to the simple phylogenies resulting from inappropriate sampling. Here, we focus on the taxonomy of a small wood-inhabiting fungal genus *Hyphodermella*. With the most comprehensive sampling to date, the phylogenetic position of *Hyphodermella* within *Phanerochaetaceae* is rearranged by employing the same ITS and nLSU regions as in previous studies and also the ITS, nLSU, *rpb1*, *rpb2* and *tef1α* regions. Three species are excluded from *Hyphodermella*: *H. poroides* is placed in a newly introduced monotypic genus *Pseudohyphodermella*, while *H. aurantiaca* and *H. zixishanensis* are transferred to *Roseograndinia*. *Hyphodermella suiae* is described as a new species from South China and Vietnam. Keys to eight species in *Hyphodermella* and five in *Roseograndinia* are provided. Beyond solving the taxonomic issue of *Hyphodermella* itself, the current study also aims to suggest that all fungal taxonomists especially beginners should keep in mind to sample as many comprehensive taxa as possible in phylogenetic analyses.

**Supplementary Information:**

The online version contains supplementary material available at 10.1186/s43008-023-00116-7.

## INTRODUCTION

**Fig. 1:**
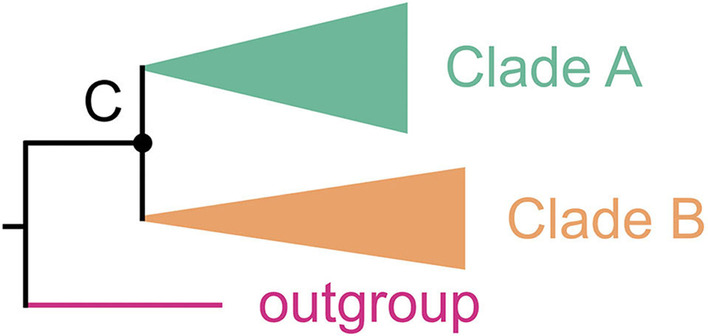
A schematic illustration of the ‘simple phylogeny’ resulted from inappropriate sampling in a bad practice of phylogenetic analyses. Whatever the statistical support at the node C is high or not, species in Clade A is not always congeneric with species in Clade B

Despite being one of the most species-rich life forms, *Fungi* are poorly documented with more than 90% of estimated species (2.2 to 3.8 million species) awaiting formal description (Hawksworth and Lücking 2017). To enlarge the knowledge of fungal diversity, more than one thousand species have been newly introduced each year during the last decades (Dai et al. 2015; Hawksworth and Lücking 2017; Niskanen et al. 2018). Above the species level, genus is a special and crucial taxonomic rank compared with other ranks under the binomial nomenclature system, because a species has to be placed in a certain genus but may be not assigned in any certain higher rank than genus. Although molecular phylogenies are helpful to determine the generic position of fungal species, the placements are sometimes incorrect due to the use of simple phylogenies resulted from inappropriate sampling in a bad practice of phylogenetic analyses. Here, a simple phylogeny is defined to sample only targeted species but not closely related outgroup taxa; in this way, the generic circumscription cannot be reliably delimited (Fig. 1). In contrast, a “good” genus can only be accurately delimited by sampling more related taxa to the targeted species. Indeed, increased taxon sampling has long been known as an efficient method to reduce error signals in phylogenetic analyses (Zwickl and Hillis 2002; Prasanna et al. 2020).

To clearly present the results of simple phylogenetic studies in fungal taxonomy and the resulting incorrect generic placements of taxa, two examples recently dealt with by us are briefly summarized here. One is the incorrect placements at the generic level of two species originally placed in *Heteroradulum*, viz. *H. yunnanense* (with the wrong masculine gender as ‘*yunnanensis*’; Guan et al. 2020) and *H. niveum* (Li et al. 2022a). In Guan et al. (2020), several taxa of *Heteroradulum* were selected as the only ingroup and *H. yunnanense* was placed at the basal position within the so-called *Heteroradulum* lineage; actually, this simple phylogeny cannot determine whether *H. yunnanense* should be the member of *Heteroradulum* or not. With the help of a more comprehensive sampling, a later phylogenetic analysis clearly separated *H. yunnanense* from *Heteroradulum* and thus excluded it from this genus (Li et al. 2022b).

Similarly, according to a simple phylogeny (Li et al. 2022a: Fig. 2) adopted from Guan et al. (2020), the new species *Heteroradulum niveum* was further incorrectly placed in *Heteroradulum* (Li et al. 2022a). Even worse, the accompanying phylogeny in that paper (Li et al. 2022a: Fig. 1) did not cluster *H. niveum* with other species of *Heteroradulum* with reliable statistical support at all. In contrast, Liu et al. (2022b) thoroughly explored the phylogenetic relationships among *Heteroradulum* and its close genera, which resulted in a new genus *Alloexidiopsis* for the clade composed of *H. yunnanense* and *H. niveum*.

Another example is two species originally placed in *Trechispora* (Zong et al. 2021) and then in *Brevicellicium* (Liu et al. 2022c). In Zong et al. (2021), newly describing *Trechispora daweishanensis* and *T. xantha*, the first phylogeny did not recover the monophyly of *Trechispora* with these two species, while the second one simply including taxa only from *Trechispora* as the ingroup clustered the two species with *T. yunnanensis* and separated them from additional species of *Trechispora*. As first noted by Chikowski et al. (2020) and then confirmed by Liu et al. (2022a), the ITS and nLSU sequences from specimens of *T. yunnanensis* (Xu et al. 2019) actually represent different species from *Trechisporales* and *Hymenochaetales*, respectively, and thus the phylogenetic position of *T. yunnanensis* itself is doubtful. Liu et al. (2022c) recognized the incorrect generic placements of *T. daweishanensis* and *T. xantha* by Zong et al. (2021), and transferred these two species to *Brevicellicium*. However, the phylogeny supporting these transfers was also on the basis of a simple phylogeny (Liu et al. 2022c: Fig. 1), in which these two species also clustered together with species of *Brevicellicium* but occupied a separated position. By sampling the most comprehensive range of taxa in *Trechisporales* available to date, the phylogeny in Liu et al. (2022a) clarified these two species placing them outside of both *Trechispora* and *Brevicellicium*, and in a new genus, *Allotrechispora*.

Besides the examples of *Heteroradulum*, *Trechispora*, and *Brevicellicium* having been dealt with (Li et al. 2022b; Liu et al. 2022a, b), similar incorrect placements also exist in other genera. In the current study, we focus on the genus *Hyphodermella*, in which two recently collected specimens from tropical Asia are identified.

*Hyphodermella* was erected as a monotypic genus for *H. corrugata* (Eriksson and Ryvarden 1976). Besides the generic type, another eight species are accepted in this genus within *Phanerochaetaceae* (Gilbertson et al. 2001; Melo and Hjortstam 2003; Nakasone 2008; Duhem 2010; Telleria et al. 2010; Duhem and Buyck 2011; Zhao et al. 2017; Wang and Zhao 2020; Wang et al. 2021a). Within *Hyphodermella*, the generic placement of *H. poroides* is questionable. *Hyphodermella poroides* was described according to a simple phylogeny that placed this species in a basal position within a clade also comprising *H. corrugata* and *H. rosae* (Zhao et al. 2017). Besides the uncertain phylogenetic position, the poroid hymenophoral surface also makes *H. poroides* distinguished from other species of *Hyphodermella* (Zhao et al. 2017). Although macrofungal species producing various hymenophoral configurations commonly can be placed in the same genus (Wang et al. 2021b; Li et al. 2022b; Liu et al. 2022a), in this case it is obvious, not as stated in the Abstract by the authors: “Both morphological and molecular evidences confirmed the placement of the new species in *Hyphodermella*.” (Zhao et al. 2017). Chen et al. (2021) recently performed a much more comprehensive phylogenetic analysis than that of Zhao et al. (2017) which clearly revealed the separation of *H. poroides* from *Hyphodermella* (Chen et al. 2021: Fig. 3), but they did not make any taxonomic change possibly due to their focusing mainly on other taxonomic issues. Around the publication time of Chen et al. (2021), another two new species, viz. *H. aurantiaca* and *H. zixishanensis* were separately described in *Hyphodermella* by the same research group, although the related phylogenies never confirmed their close relationship with *Hyphodermella* (Wang and Zhao 2020; Wang et al. 2021a).

When examining our specimens of *Hyphodermella*, we also explored the phylogenetic relationship of this genus via the most comprehensive sampling available to date. Accordingly, one genus, one species and three combinations are newly proposed. Beyond the taxonomic issue of *Hyphodermella*, we also aim to provide a standard to better phylogenies in future taxonomic studies.

## MATERIALS and METHODS

### Morphological examination

The studied specimens are preserved at the Fungarium, Institute of Microbiology, Chinese Academy of Sciences (HMAS), Beijing, China. The hymenophoral surfaces of basidiomes were examined with a Leica M125 stereomicroscope (Wetzlar, Germany) at a magnification of up to 100 × . The microscopic characters were observed with an Olympus BX43 light microscope (Tokyo, Japan) at magnifications up to 1000 × . The microscopic procedure followed Yu et al. (2021). Basidiome sections were prepared with Cotton Blue (CB), Melzer’s reagent, and 5% potassium hydroxide (KOH). All measurements were made from sections in CB. When presenting the variation of basidiospore sizes, 5% of the measurements were excluded from each end of the range and are given in parentheses. Drawings were made with the aid of a drawing tube. In the morphological description, L = mean basidiospore length (arithmetic average of all measured basidiospores), W = mean basidiospore width (arithmetic average of all measured basidiospores), Q = variation in the L/W ratios between the studied specimens, and (a/b) = the number of measurements (a) from a given number (b) of specimens.

### Molecular sequencing

A small piece of basidiome was taken for DNA extraction using a CTAB rapid plant genome extraction kit-DN14 (Aidlab Biotechnologies, Beijing). Then, the crude DNA was used as templates for PCR amplifications of ITS, nLSU, *rpb1*, *rpb2* and *tef1α* regions with the primer pairs ITS5/ITS4 (White et al. 1990), LROR/LR7 (Gardes and Bruns 1993), RPB1-Af/RPB1-Cr (Matheny et al. 2002), RPB2-f5F/RPB2-b7.1R (Liu et al. 1999; Matheny 2005) and 983F/1567R (Rehner and Buckley 2005), respectively. The PCR procedure was as follows: initial denaturation at 95 °C for 3 min, followed by 34 cycles at 94 °C for 40 s, 57.2 °C for 45 s and 72 °C for 1 min, and a final extension at 72 °C for 10 min for ITS and *tef1α* regions; initial denaturation at 94 °C for 1 min, followed by 35 cycles at 94 °C for 30 s, 48 °C 1 min and 72 °C for 1.5 min, and a final extension of 72 °C for 10 min for nLSU region; initial denaturation at 94 °C for 2 min, followed by 10 cycles at 94 °C for 45 s, 60 °C for 45 s (minus 1 °C per cycle) and 72 °C for 1.5 min, then followed by 36 cycles at 94 °C for 45 s, 53 °C for 1 min and 72 °C for 1.5 min, and a final extension of 72 °C for 10 min for *rpb1* and *rpb2* regions. The PCR products were sequenced with the same primers as those used in PCR amplification at the Beijing Genomics Institute, Beijing, China. All newly generated sequences were deposited in GenBank (https://www.ncbi.nlm.nih.gov/genbank/; Table 1).

**Table 1:** Species and sequences used in phylogenetic analyses

Species name	Collection No	Collection locality	Collection date	Accession No				
				ITS	nLSU	*rpb1*	*rpb2*	*tef1α*
*Alboefibula bambusicola*	Chen 2304	China: Taiwan	27 Jun 2014	MZ636926	MZ637091	MZ748355	OK135980	MZ913590
*Alboefibula bambusicola*	Wu 1209-26	China: Taiwan	15 Sept 2012	MZ636927	MZ637092			
*Alboefibula gracilis*	Wu 1809-106	China: Guangxi	10 Sept 2018	MZ636929	MZ637094	MZ748357	OK135982	MZ913591
*Alboefibula gracilis*	Wu 1809-152	China: Guangxi	10 Sept 2018	MZ636930	MZ637095			
*Bjerkandera adusta*	HHB-12826-Sp	USA: Alaska		KP134983	KP135198			
*Byssomerulius corium*	FP-102382	USA: Wisconsin		KP135007	KP135230			
*Candelabrochaete africana*	FP-102987-Sp	USA: Puerto Rico		KP135294	KP135199			
*Ceriporia purpurea*	KKN 223	USA: Arizona		KP135044	KP135203			
*Ceriporia viridans*	GC 1708-211	China: Yunnan		LC427027	LC427049			
*Climacodon septentrionalis*	AFTOL-767	Unknown		AY854082	AY684165			
*Crepatura ellipsospora*	CLZhao 1260	China: Yunnan	22 Apr 2017	MK343693	MK343697			
*Crepatura ellipsospora*	CLZhao 1265	China: Yunnan	22 Apr 2017	MK343692	MK343696			
*Crystallicutis damiettensis*	UN63	Egypt: Kafr El-Sheikh, Baltim	14 Feb 2014	MW508515	MW508515			
*Crystallicutis serpens*	HHB-15692-Sp	USA: Alaska		KP135031	KP135200			
*Donkia pulcherrima*	GC 1707-11	China: Taiwan	23 Jul 2017	LC378994	LC379152	LC379157	LC387351	LC387371
*Donkia pulcherrima*	Gothenburg-2022	Austria		KX752591	KX752591			
*Efibulella deflectens*	FCUG 1568	Sweden		AF141619	AF141619			
*Emmia latemarginata*	CBS 436.48	Canada: British Columbia		MH856427	MH867973			
*Gelatinofungus brunneus*	Wu 1207-162	China: Taiwan	10 Jul 2012	MZ636978	MZ637139	MZ748366	OK136005	MZ913615
*Gelatinofungus brunneus*	Wu 1207-163	China: Taiwan	10 Jul 2012	MZ636979	MZ637140			
*Geliporus exilisporus*	Dai 2172	China: Liaoning	25 Sept 1995	KU598211	KU598216			
*Geliporus exilisporus*	GC 1702-15	China: Taiwan	19 Feb 2017	LC378995	LC379153	LC379158	LC387352	LC387372
*Gloeoporus conchoides*	BZ-2896	Belize		MG572757	MG572741			
*Gloeoporus pannocinctus*	L-15726-Sp	USA: New York		KP135060	KP135214			
*Hapalopilus eupatorii*	Dammrich 10744	Germany		KX752620	KX752620			
*Hapalopilus percoctus*	H 7008581	Botswana		KX752597	KX752597			
*Hapalopilus rutilans*	CBS 422.48	Canada: Ontario		MH856419	MH867966			
*Hydnophlebia chrysorhiza*	FD-282	USA: Florida		KP135338	KP135217			
*Hyphoderma litschaueri*	FP-101740-Sp	USA: Wisconsin		KP135295	KP135219			
*Hyphoderma mutatum*	HHB-15479-Sp	USA: Alaska		KP135296	KP135221			
*Hyphodermella corrugata*	MA-Fungi 24238	Portugal	28 Apr 1989	FN600378	JN939586			
*Hyphodermella corrugata*	MA-Fungi 5527	Morocco	20 Jun 1982	FN600372	JN939597			
*Hyphodermella corrugata*	MA-Fungi 61395	France	31 Oct 1998	FN600380	JN939584			
*Hyphodermella pallidostraminea*	LE 286968	Russia: Jewish Autonomous Oblast	24 Aug 2009	OK138912	OK138911			
*Hyphodermella rosae*	FP-150552	USA: Hawaii		KP134978	KP135223			
*Hyphodermella rosae*	GC 1608-2	Japan		MZ636987	MZ637148	MZ748411	OK135983	MZ913592
*Hyphodermella suiae*	LWZ 20190613-54	China: Guangdong	13 Jun 2019	**ON614149**	**ON614151**	**OP698136**	**OP698133**	
*Hyphodermella suiae*	LWZ 20191208-13	Malaysia: Kuala Lumpur	08 Dec 2019	**ON614150**			**OP698134**	**OP698135**
*Irpex lacteus*	FD-9	USA: Massachusetts		KP135026	KP135224			
*Meruliopsis albostramineus*	HHB-10729	USA: Virginia		KP135051	KP135229			
*Mycoacia fuscoatra*	HHB-10782-Sp	USA: Wisconsin		KP135365	KP135265			
*Odontoefibula orientalis*	Wu 0805-59	China: Taiwan	22 May 2008	LC363488	LC363493			
*Odontoefibula orientalis*	Wu 0910-57	China: Beijing	14 Oct 2009	LC363490	LC363495	LC363501	LC387362	LC387381
*Oxychaete cervinogilva*	Dmitry Schigel 5216	Australia		KX752596	KX752596			
*Phaeophlebiopsis caribbeana*	HHB-6990	USA: Florida		KP135415	KP135243			
*Phaeophlebiopsis peniophoroides*	FP-150577	USA: Hawaii		KP135417	KP135273			
*Phanerina mellea*	Dai 9667	China: Hainan	26 May 2008	JX623933	JX644058			
*Phanerina mellea*	WEI 17-224	China: Taiwan	11 Jun 2017	LC387333	LC387340			
*Phanerochaete alnea*	Spirin 8829a	Canada: Alberta		KX538925				
*Phanerochaete australis*	HHB-7105-Sp	USA: Florida		KP135081	KP135240			
*Phanerochaete burtii*	HHB-4618-Sp	USA: Florida		KP135117	KP135241			
*Phanerochaete canobrunnea*	CHWC 1506-66	China: Taiwan	23 Jun 2015	LC412095	LC412104			
*Phanerochaete ericina*	HHB-2288	USA: North Carolina		KP135167	KP135247			
*Phanerochaete fusca*	Wu 1409-161	China: Hubei	19 Sept 2014	LC412098	LC412105			
*Phanerochaete laevis*	HHB-15519-Sp	USA: Alaska		KP135149	KP135249			
*Phanerochaete porostereoides*	He 1908	China: Shannxi	11 Sept 2013	KX212218	KX212222			
*Phanerochaete pseudomagnoliae*	PP-25	South Africa		KP135091	KP135250			
*Phanerochaete rhodella*	FD-18	USA: Massachusetts		KP135187	KP135258			
*Phanerodontia chrysosporium*	HHB-6251-Sp	USA: Arizona		KP135094	KP135246			
*Phlebia centrifuga*	HHB-9239-Sp	USA: Michigan		KP135380	KP135262			
*Phlebia radiata*	AFTOL-484	Unknown		AY854087	AF287885			
*Phlebiopsis crassa*	KKN-86-Sp	USA: Arizona		KP135394	KP135215			
*Phlebiopsis flavidoalba*	FD-263	USA: Florida		KP135402	KP135271			
*Phlebiopsis gigantea*	FP-70857-Sp	USA: Georgia		KP135390	KP135272			
*Phlebiopsis pilatii*	Spirin 5048	Russia		KX752590	KX752590			
*Pirex concentricus*	Kropp160Bup6-R	USA: Oregon		KP134985				
*Pirex concentricus*	OSC-41587	USA: Oregon		KP134984	KP135275	KP134843	KP134940	
*Porostereum spadiceum*	Wu 9708-104	China			DQ679918			
*Pseudohyphodermella poroides*	Dai 10848	China:Hainan	11 May 2009	KX008368	KX011853			
*Pseudohyphodermella poroides*	Dai 12045	China: Hainan	25 Nov 2010	KX008367	KX011852			
*Quasiphlebia densa*	WEI 17-057	USA: Georgia	23 Apr 2017	MZ637066	MZ637265	MZ748410	OK135986	MZ913630
*Quasiphlebia densa*	Wu 9304-33	Taiwan	13 Apr 1993	MZ637067	MZ637266	MZ748409		MZ913629
*Rhizochaete brunnea*	MR11455	Argentina	23 Mar 1998	AY219389	AY219389			
*Rhizochaete fouquieriae*	KKN121 sp	USA: Arizona		KY948786	KY948858			
*Rhizochaete radicata*	FD-123	USA: Massachusetts		KP135407	KP135279			
*Riopa metamorphosa*	JV 0511/5	Czech Republic		KX752613	KX752613			
*Riopa pudens*	Cui 3238	China	22 Oct 2005	JX623931	JX644060			
*Roseograndinia aurantiaca*	CLZhao 10487	China: Yunnan	10 Jan 2019	MW209023	MW209012			
*Roseograndinia aurantiaca*	CLZhao 10491	China: Yunnan	10 Jan 2019	MW209024	MW209013			
*Roseograndinia jilinensis*	Wu 1307-132	China: Jilin	14 Jul 2013	MZ637076	MZ637274	MZ748412	OK135984	MZ913631
*Roseograndinia jilinensis*	Wu 1307-137	China: Jilin	14 Jul 2013	MZ637077	MZ637275	MZ748413	OK135985	MZ913632
*Roseograndinia minispora*	WEI 18-508	China: Taiwan	05 Nov 2018	MZ637078	MZ637276			
*Roseograndinia minispora*	WEI 18-511	China: Taiwan	05 Nov 2018	MZ637079	MZ637277			
*Roseograndinia zixishanensis*	CLZhao 7206	China: Yunnan	01 Aug 2018	MZ305280	MZ305289			
*Roseograndinia zixishanensis*	CLZhao 7718	China: Yunnan	01 Aug 2018	MZ305285	MZ305293			
*Scopuloides rimosa*	HHB-7042-Sp	USA: Florida		KP135350	KP135282			
*Terana caerulea*	FP-104073	USA: Maryland		KP134980	KP135276			

Newly generated sequences are in bold

### Phylogenetic analyses

Besides the newly generated sequences, additional molecular sequences were downloaded from GenBank for the phylogenetic analysis (Table 1). Two datasets were assembled to explore the phylogenetic position of our specimens in *Hyphodermella* and, more importantly, the phylogenetic relationship among *Hyphodermella* and related genera within *Phanerochaetaceae*. For the dataset of the combined ITS and nLSU regions, genera represented mostly by generic types in *Phanerochaetaceae* as well as *Irpicaceae* and *Meruliaceae* were comprehensively sampled as ingroup taxa. *Hyphoderma litschaueri*, *H. mutatum* and *Candelabrochaete africana* were selected as outgroup taxa (Chen et al. 2021). For the dataset of combined ITS, nLSU, *rpb1*, *rpb2* and *tef1α* regions, genera phylogenetically close to our specimens were further sampled as ingroup taxa and *Gelatinofungus brunneus* was selected as the outgroup taxon according to the topology resulting from the previous two-locus dataset. ITS, nLSU, *rpb1*, *rpb2* and *tef1α* regions were separately aligned using MAFFT 7.110 (Katoh and Standley 2013) under the G-INS-i option (Katoh et al. 2005), and the ambiguous regions of the alignments were trimmed using trimAl v1.2 under default parameters (Capella-Gutiérrez et al. 2009). Firstly, the resulting alignments for each locus were separately subjected to phylogenetic analyses, and no conflict in main lineages of our targeted taxonomic groups was observed from each other (data not shown). Then, the resulting alignments were concatenated as two alignments corresponding to the two datasets (Additional file 1: Alignment S1, Additional file 2: Alignment S2). The ITS region in these two alignments were further divided into ITS1, 5.8S and ITS2 subregions using ITSx 1.1.2 (Bengtsson-Palme et al. 2013) for separate model selection of phylogenetic analyses.

The maximum likelihood (ML) algorithm was performed using IQ-tree v2.1.2 (Minh et al. 2020), which implements automatic substitution model selection for each locus in ModelFinder (Kalyaanamoorthy et al. 2017) assessing nodal support determined by ultrafast bootstrapping (BS) with 10,000 replicates. The Bayesian inference (BI) algorithm was performed using MrBayes 3.2 (Ronquist et al. 2012). jModelTest 2 was used to estimate the best-fit evolutionary models of all loci separately for the BI algorithm under the corrected Akaike information criterion (Guindon and Gascuel 2003; Posada 2008). A discrete gamma distribution was used to model evolutionary rate differences among sites (four categories, + G). In the BI algorithm, two independent runs, each with four chains of one million generations and starting from random trees, were employed; trees were sampled every 1000th generation, of which the first 25% were removed as burn-in and the other 75% were retained for constructing a 50% majority consensus tree and calculating Bayesian posterior probabilities (BPPs). Tracer 1.5 (http://tree.bio.ed.ac.uk/software/tracer/) was used to judge whether chains converged.

## RESULTS

**Fig. 2:**
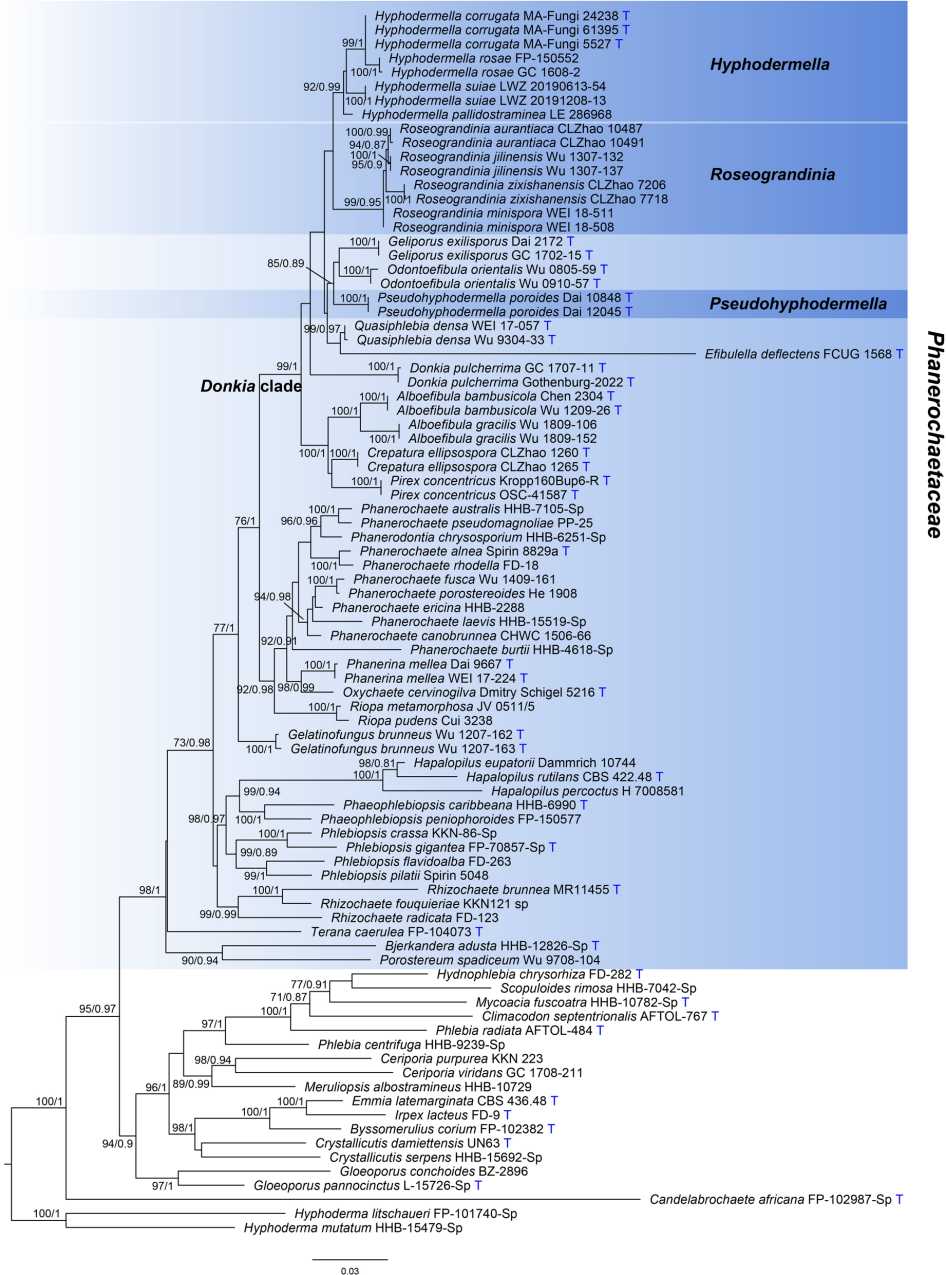
Phylogenetic relationships among *Hyphodermella* and related genera inferred from ITS and nLSU regions. The topology was generated from the maximum likelihood algorithm, and bootstrap values and Bayesian posterior probabilities simultaneously above 50% and 0.8, respectively, are presented at the nodes. *Phanerochaetaceae* is indicated by the background in blue color, and the three genera related to *Hyphodermella* in darker blue color. The generic type species are indicated by the blue character T at the end of tip labels

Seven new sequences were generated from our specimens for this study (Table 1). The concatenated alignment of ITS and nLSU regions included 1643 characters with 311 parsimony-informative ones from 87 collections representing 68 species. For the ML algorithm, the best-fit partitioned models were determined as TVM + F + I + I + R4 for ITS1, GTR + F + I + I + R3 for both 5.8S and nLSU, and GTR + F + R4 for ITS2. For the BI algorithm, K80 + G, JC, JC and GTR + I + G were estimated as the best-fit partitioned models for the partitions of ITS1, 5.8S, ITS2 and nLSU, respectively. All chains in BI converged after ten million generations, which is indicated by the effective sample sizes of all parameters above 200 and the potential scale reduction factors close to 1.000. ML and BI algorithms construct similar topologies that differed only at several poorly supported nodes. The topology resulted from the ML algorithm is shown along with BS values more than 50% and BPPs more than 0.8 at the nodes (Fig. 2). In this phylogeny, the sampled species of *Hyphodermella* are separated in three lineages within the *Donkia* clade of *Phanerochaetaceae* (Fig. 2). The core lineage comprises the generic type *Hyphodermella corrugata*, *H. pallidostraminea* and *H. rosae* (BS = 92%, BPP = 0.99). In addition, two newly sequenced specimens, viz. LWZ 20190613-54 from Guangdong, China and LWZ 20191208-13 from Malaysia fall within the core lineage of *Hyphodermella*, and are separated from other species in this lineage. *Hyphodermella aurantiaca* and *H. zixishanensis* grouped together with two species of *Roseograndinia* (BS = 99%, BPP = 0.95). *Hyphodermella poroides* forms an independent lineage from other genera and species (BS = 100%, BPP = 1).

**Fig. 3:**
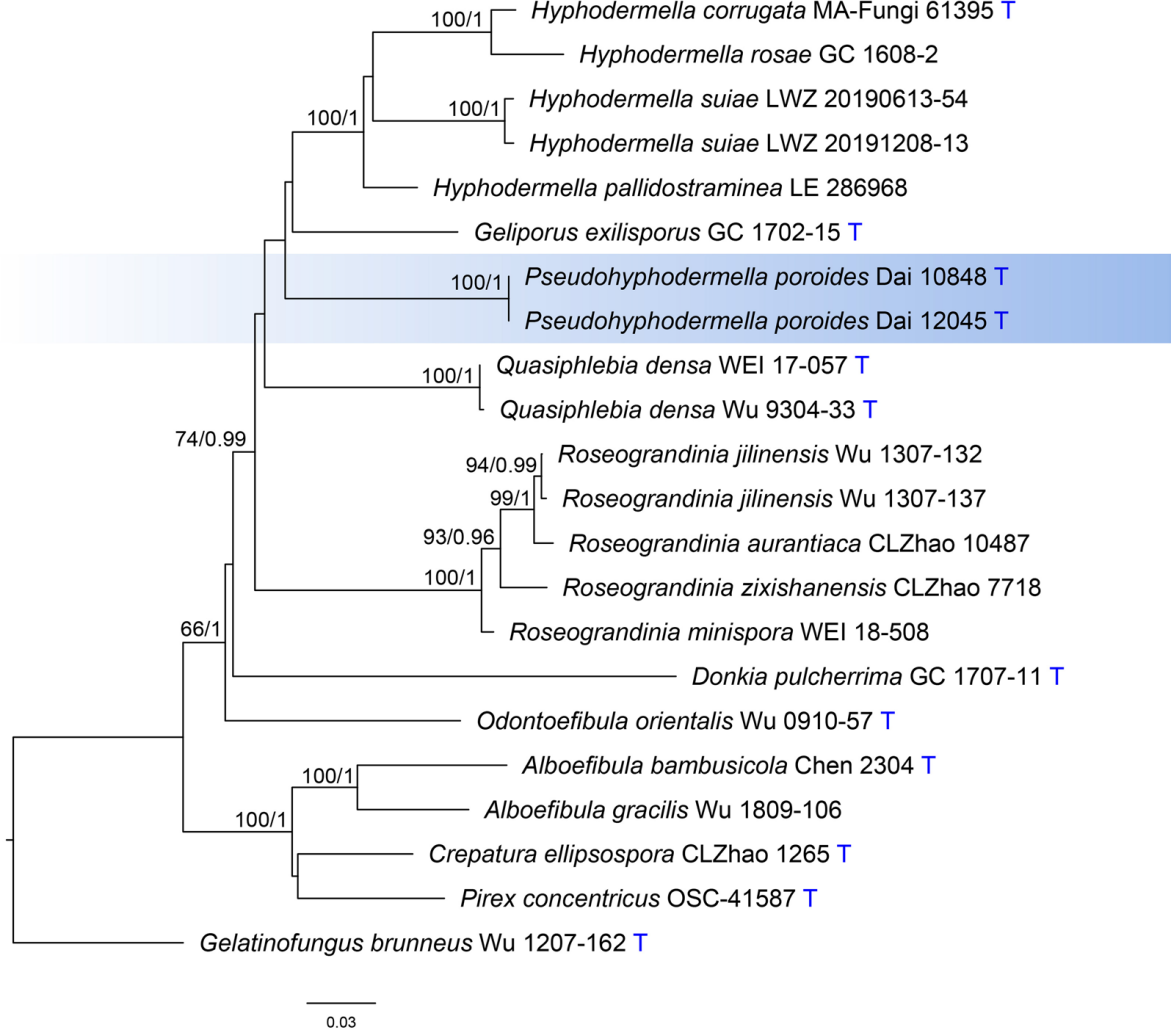
Phylogenetic relationships among *Hyphodermella* and related genera inferred from ITS, nLSU, *rpb1*, *rpb2* and *tef1α* regions. The topology was generated from the maximum likelihood algorithm, and bootstrap values and Bayesian posterior probabilities simultaneously above 50% and 0.8, respectively, are presented at the nodes. *Pseudohyphodermella* is indicated by the background in blue color. The generic type species are indicated by the blue character T at the end of tip labels

The concatenated alignment of ITS, nLSU, *rpb1*, *rpb2* and *tef1α* regions included 4550 characters with 882 parsimony-informative ones from 22 collections representing 18 species. For the ML algorithm, the best-fit partitioned models were determined as TPM2u + F + I + I + R2 for ITS1, TN + F + R2 for both 5.8S and nLSU, TIM + F + I + I + R2 for ITS2, GTR + F + I + I + R3 for both *rpb1* and *rpb2*, and TIM2 + F + I + I + R2 for *tef1α*. For the BI algorithm, SYM + G, K80 and HKY + I + G were estimated as the best-fit partitioned models for the partitions of ITS1, 5.8S and ITS2, respectively, and GTR + I + G for all of nLSU, *rpb1*, *rpb2* and *tef1α*. All chains in BI converged after one million generations, which is indicated by the effective sample sizes of all parameters above 200 and the potential scale reduction factors close to 1.000. ML and BI algorithms construct similar topologies that differed only at several poorly supported nodes. The topology resulted from the ML algorithm is shown along with BS values more than 50% and BPPs more than 0.8 at the nodes (Fig. 3). Like the phylogeny inferred from the dataset of combined ITS and nLSU regions (Fig. 2), this five-locus based phylogeny also recovered the sampled species of *Hyphodermella* in three independent lineages and the distinct position of the two newly sequenced specimens within the core lineage (Fig. 3).

In association with morphological characters, the two newly sequenced specimens are described as a new species of *Hyphodermella*, a new genus is erected for *H. poroides*, and *H. aurantiaca* and *H. zixishanensis* are transferred to *Roseograndinia.*

## TAXONOMY

**Fig. 4:**
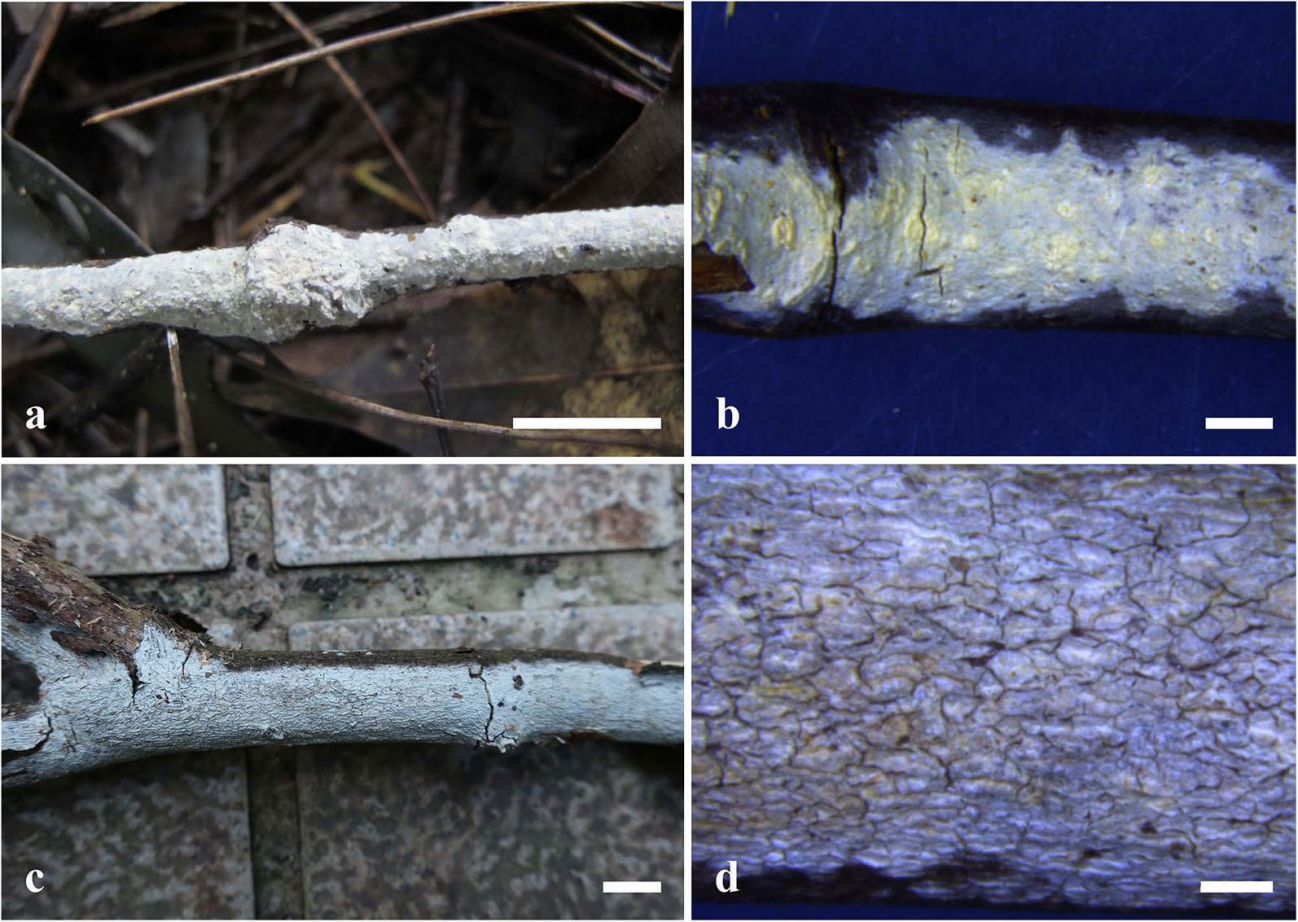
Basidiomes of *Hyphodermella suiae* (**a**–**d**) in general and detailed views. **a**, **b** LWZ 20190613-54 (holotype); **c**, **d** LWZ 20191208-13 (paratype). Bars: **a**, **c** = 1 cm; **b**, **d** = 2 mm

**Fig. 5:**
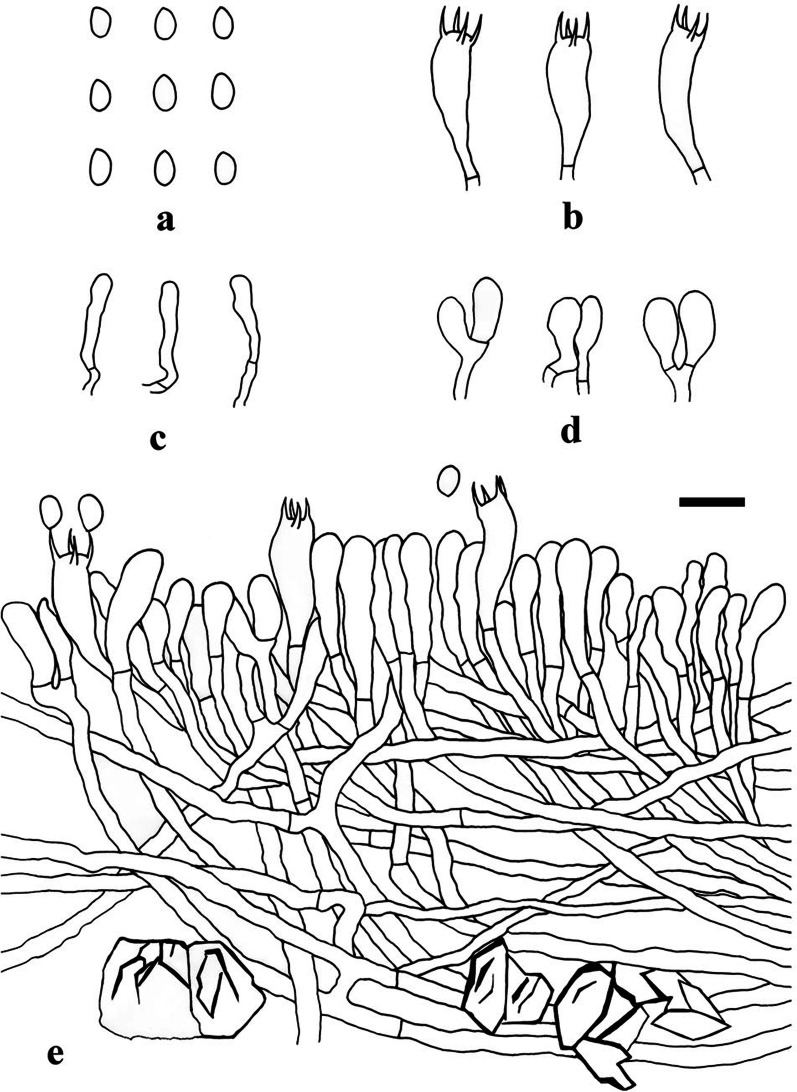
Microscopic structures of *Hyphodermella suiae* (drawn from LWZ 20190613-54, holotype). **a** Basidiospores. **b** Basidia. **c** Cystidioid hyphal ends. **d** Basidioles. **e** A vertical section through basidiomes. Bars: 10 μm

***Hyphodermella suiae*** Shan Shen, S.L. Liu & L.W. Zhou, **sp. nov. (**Figs. 4, 5**)**

MycoBank: MB 848641

*Etymology*: *suiae* (Lat.), in memory of the Chinese mycologist Hong-Yan Su (苏 鸿雁), who was a professor in Dali University and kindly helped the corresponding author in many ways; she passed away on 3 May 2022 during the preparation of the current paper at the age of 55 years.

*Diagnosis*: Distinguished from other species of *Hyphodermella* by the small basidiospores.

*Type*: **China:** *Guangdong*: Ruyuan County, Nanling National Forest Park, on fallen angiosperm twig, 13 Jun 2019, *Li-Wei Zhou*, LWZ 20190613-54 (HMAS 287394—holotype).

*Description*: *Basidiomes* annual, resupinate, adnate, adherent, without odor or taste, leathery when fresh, up to 2.5 cm wide, 15 cm long and 100–150 μm thick. Hymenophoral surfaces smooth to tuberculate, shaped with the substrate shape partly, white to pale buff when fresh, becoming darker buff pale and cracking when drying. Margin distinct, white.

*Hyphal system* monomitic; generative hyphae with simple septa, thin-walled, 2.5–4 μm (*n* = 40/2) diam, branched, acyanophilous, inamyloid, indextrinoid, interwoven in subhymenium, more or less regularly arranged in subiculum; tissue unchanged in KOH. *Basidia* clavate, with four sterigmata and a basal simple septum, 20–25 × 5.5–6.5 μm (*n* = 40/2); basidioles dominant, in shape similar to basidia, but slightly smaller. *Cystidia* and cystidioles absent; cystidioid hyphal ends occasionally present, narrow clavate, thin-walled. Crystals present among hyphae, rhomboidal. *Basidiospores* ellipsoid, hyaline, thin-walled, smooth, inamyloid, indextrinoid, acyanophilous, (4.1–)4.2–5.2(–5.3) × 3.1–3.9(–4) µm, L = 4.81 μm, W = 3.42 μm, Q = 1.39–1.44 (*n* = 60/2).

*Additional specimen examined*: **Malaysia:** *Kuala Lumpur*: KL Forest Eco park, on fallen angiosperm twig, 8 Dec 2019, *Li-Wei Zhou*, LWZ 20191208-13 (HMAS 287395).

*Notes*: *Hyphodermella suiae* is similar to *H. brunneocontexta* in the smooth to tuberculate hymenophoral surface and the size of basidiospores. However, the hyphae of *H. brunneocontexta* in subiculum are thick-walled and brown (Duhem and Buyck 2011), while *H. suiae* has thin-walled, hyaline hyphae. In addition, *H. suiae* differs in having smaller basidiospores than the three species of *Hyphodermella* sampled in the current phylogenetic analysis, viz. *H. corrugate* (7–10 × 4–6 μm, Eriksson and Ryvarden 1976), *H. pallidostraminea* (5.4–6.6 × 3–3.5 μm, Crous et al. 2021), and *H. rosae* (6–8 × 4.3–5 μm, Nakasone 2008).

***Pseudohyphodermella*** Shan Shen, S.L. Liu & L.W. Zhou, **gen. nov**.

MycoBank: MB 848651

*Etymology*: *Pseudohyphodermella* (Lat.), referring to the incorrect placement of the generic type in *Hyphodermella*.

*Diagnosis*: Distinguished from other genera in *Phanerochaetaceae* by the annual, resupinate basidiomes, a poroid hymenophore configuration, tissues unchanged in KOH, absence of cystidia, and broadly ellipsoid basidiospores.

*Type*: *Pseudohyphodermella poroides* (Y.C. Dai & C.L. Zhao) Shan Shen et al. 2023.

*Description*: *Basidiomes* annual, resupinate, effused. Hymenophoral surface poroid, cream to orange. *Hyphal system* monomitic; generative hyphae with simple septa, hyaline, thin-walled, wider in subiculum than in trama. *Cystidia* absent. *Basidia* clavate, hyaline, thin-walled, with four sterigmata and a basal simple septum. *Basidiospores* broadly ellipsoid, hyaline, thin-walled, smooth, inamyloid, indextrinoid, acyanophilous.

*Notes*: Within the *Donkia* clade of *Phanerochaetaceae*, the poroid hymenophoral surface makes *Pseudohyphodermella* and *Geliporus* distinct from other genera. Moreover, the tissues of *Pseudohyphodermella* do not change in KOH and the basidiospores are broadly ellipsoid (Zhao et al. 2017), while *Geliporus* has tissues that darken in KOH and cylindric to oblong-ellipsoid basidiospores (Yuan et al. 2017). In addition, *Phanerina* and *Riopa* fall outside the *Donkia* clade but within *Phanerochaetaceae* but also resemble *Pseudohyphodermella* in having resupinate basidiomes with a poroid hymenophoral surface; however, these two genera differ in the presence of cystidia and curved cylindrical to narrow ellipsoid basidiospores (Miettinen et al. 2016).

***Pseudohyphodermella poroides*** (Y.C. Dai & C.L. Zhao) Shan Shen, S.L. Liu & L.W. Zhou, **comb. nov.**

MycoBank: MB 848652

*Basionym*: *Hyphodermella poroides* Y.C. Dai & C.L. Zhao, *Mycoscience* **58**: 454 (2017).

*Notes*: *Pseudohyphodermella poroides* was originally described in *Hyphodermella* with a simple phylogeny as reference (Zhao et al. 2017). Although this species shares some morphological characters with *Hyphodermella*, such as a monomitic hyphal system with simple-septate generative hyphae and absence of cystidia, its poroid hymenophoral surface makes it distinguished from other species of *Hyphodermella*. Chen et al. (2021) first revealed the separation of *H. poroides* from *Hyphodermella* from a phylogenetic perspective. The current phylogeny (Fig. 2) further confirms the independence of *H. poroides* from all known genera and species. Therefore, a new genus *Pseudohyphodermella* is erected for this species, and *H. poroides* is accordingly transferred as *P. poroides.*

***Roseograndinia aurantiaca*** (C.L. Zhao) Shan Shen, S.L. Liu & L.W. Zhou, **comb. nov.**

MycoBank: MB 848653

*Basionym*: *Hyphodermella aurantiaca* C.L. Zhao, *Ann. bot. fenn.* **58**: 65 (2020).

*Notes*: *Hyphodermella aurantiaca* was recently described as a new species; however, the original simple phylogenies inferred from the nLSU region and a combination of ITS and nLSU regions did not provide reliable statistical support for the taxonomic position of this species in *Hyphodermella* (Wang and Zhao 2020). With our more comprehensive sampling, the current phylogeny strongly supports *H. aurantiaca* being separated from *H. corrugata* the type species of *Hyphodermella* and grouping together with species of *Roseograndinia* (BS = 99%, BPP = 0.95; Fig. 2). Morphologically, the combination of rose-colored basidiomes with a smooth to tuberculate hymenophoral surface, absence of cystidia and ellipsoid basidiospores makes *H. aurantiaca* consistent with the concept of *Roseograndinia* sensu Chen et al. (2021). Accordingly, *H. aurantiaca* is transferred as *Roseograndinia aurantiaca*.

***Roseograndinia zixishanensis*** (C.L. Zhao) Shan Shen, S.L. Liu & L.W. Zhou, **comb. nov.**

MycoBank: MB 848654

*Basionym*: *Hyphodermella zixishanensis* C.L. Zhao, *Nordic Jl Bot.* 38(8): e03329, 4 (2021).

*Notes*: *Hyphodermella zixishanensis* was described as a new species in *Hyphodermella* (Wang et al. 2021a, b) soon after the publication of *H. aurantiaca* (Wang and Zhao 2020). Like Wang and Zhao (2020), the simple phylogenies in Wang et al. ([Bibr CR40][Bibr CR39]) also did not reliably support the taxonomic position of this species in *Hyphodermella*. Instead, *H. zixishanensis* and *H. aurantiaca* formed a strongly supported clade (Wang et al. 2021a, b). The current phylogeny with a more comprehensive sampling strongly supports a close phylogenetic relationship between these two species and *Roseograndinia* (Fig. 2). Morphologically, *H. zixishanensis* is characterized by reddish, ceraceous basidiomes with a tuberculate hymenophoral surface and the absence of cystidia, which fits the concept of *Roseograndinia *sensu Chen et al. (2021). Therefore, *H. zixishanensis* is transferred as *Roseograndinia zixishanensis*.

### A key to all eight known species in *Hyphodermella*

**Table:** 

1 Basidiospores > 8 μm in length	2
Basidiospores < 8 μm in length	3
2 (1) Hymenophore surface orange to yellow orange; basidia > 35 μm in length	*H. corrugata*
Hymenophore surface ochraceous; basidia < 35 μm in length	*H. ochracea*
3 (1) Cystidia present	*H. maunakeaensis*
Cystidia absent	4
4 (3) Hymenophore surface odontioid	5
Hymenophore surface smooth to tuberculate	6
5 (4) Basidia suburniform to cylindric, 18–25 × 5–6.5 μm	*H. densa*
Basidia more or less clavate, 24–35 × 6–8 μm	*H. rosae*
6 (4) Hyphae thin-walled	*H. suiae*
Hyphae thick-walled, especially in subiculum	7
7 (6) Basidiomes pale yellowish; generative hyphae hyaline	*H. pallidostraminea*
Basidiomes pale grey or olive or brown; generative hyphae brown to dark brown in subiculum	*H. brunneocontexta*

### A key to all five known species in *Roseograndinia*

**Table:** 

1 Hymenophoral surface grandinioid to odontioid	2
Hymenophoral surface smooth to tuberculate	3
2 (1) Basidiospores < 3.1 μm in width, each with 1–2 oil drops	*R. jilinensis*
Basidiospores > 3.1 μm in width, without oil drops	*R. rosea*
3 (1) Basidia > 20 μm in length	*R. zixishanensis*
Basidia < 20 μm in length	4
4 (3) Basidiomes to 130 μm thick; basidiospores > 4 μm in length	*R. minispora*
Basidiomes 300–500 μm thick; basidiospores < 4 μm in length	*R. aurantiaca*

## DISCUSSION

With the most comprehensive sampling to date in the current phylogenetic analyses (Figs. 2, 3), our specimens were identified as definitely belonging in *Hyphodermella*, being described as a new species, *H. suiae*. Although the current two-locus based phylogeny (Fig. 2) and the previous phylogenies related to *Hyphodermella* (Zhao et al. 2017; Wang and Zhao 2020; Chen et al. 2021; Wang et al. 2021a) are all inferred from the ITS and nLSU regions, the relationship at the generic level will be more accurate with sampling more comprehensive taxa in phylogenetic analyses (Fig. 2; Chen et al. 2021: Fig. 3). Furthermore, the five-locus based phylogenetic analysis we performed and the resulting phylogeny (Fig. 3) further confirmed the accuracy of phylogenetic relationships among sampled species of *Hyphodermella* inferred from the ITS and nLSU regions. Accordingly, *Hyphodermella aurantiaca*, *H. poroides* and *H. zixishanensis* are all excluded from *Hyphodermella*.

*Hyphodermella poroides*, occupying an independent phylogenetic position (Figs. 2, 3), is placed in a newly introduced monotypic genus *Pseudohyphodermella*. This new genus forms a weakly supported clade with *Geliporus* and *Odontoefibula* in the two-locus based phylogeny (BS = 85%, BPP = 0.89; Fig. 2), and has no close relationship with these two genera or any other genera in the five-locus based phylogeny (Fig. 3). Therefore, the alternative options of generic delimitation instead of erecting the new monotypic genus as suggested by Vellinga et al. (2015) cannot be supported according to the current phylogenies.

*Roseograndinia* was erected as a monotypic genus for *R. rosea* (Hjortstam and Ryvarden 2005). Due to a lack of molecular sequences from the type species of the genus, *R. rosea*, the phylogenetic independence of this genus in *Phanerochaetaceae* was recovered by two morphologically similar species *R. jilinensis* and *R. minispora* (Chen et al. 2021) and we follow the taxonomic proposal by Chen et al. (2021). The current phylogenies (Figs. 2, 3) strongly support the clade comprising *H. aurantiaca*, *H. zixishanensis*, *R. jilinensis*, and *R. minispora*. Moreover, morphologically *H. aurantiaca* and *H. zixishanensis* also fit well with the concept of *Roseograndinia *sensu Chen et al. (2021). Therefore, *H. aurantiaca* and *H. zixishanensis* are transferred as *R. aurantiaca* and *R. zixishanensis* here.

We note that in the current five-locus based phylogenetic analysis, only ITS and nLSU regions are used for the *Pseudohyphodermella* lineage. That is because additional gene regions were not published when *P. poroides* was originally described (Zhao et al. 2017), and moreover, the type specimens are also unavailable for molecular sequencing as they appear to be missing from the collections of the Institute of Microbiology, Beijing Forestry University, where the types were originally deposited. Even then, according to the separation of *Hyphodermella* and *Roseograndinia* in both the two-locus and five-locus based phylogenies (Figs. 2, 3), and the separation of *Pseudohyphodermella* from *Hyphodermella* and *Roseograndinia* in the two-locus based phylogeny (Fig. 2), it is reasonable to postulate that *Pseudohyphodermella* is a *bona fide* distinct lineage from others. Taking previous phylogenies of *Hyphodermella* (Zhao et al. 2017; Wang and Zhao 2020; Chen et al. 2021; Wang et al. 2021a) into consideration together, our study indicates that the ITS and nLSU regions are enough to delimit generic circumscriptions if the related genera are comprehensively sampled in phylogenetic analyses. Namely, sampling more taxa prior to employing more genes is more crucial to explore phylogenetic relationships among genera, at least those related to *Hyphodermella*. Normally, it is better to sample all known genera in a certain family, but we recognize that sometimes this is quite difficult, if possible, when the targeted genera belong to a phylogenetically not well-resolved family. So, we suggest comprehensively sampling at least closely related genera with targeted genera in taxonomic studies in these fungi.

## CONCLUSION

In conclusion, species originally belonging to *Hyphodermella* are placed in three genera, including *Hyphodermella*, a new genus *Pseudohyphodermella*, and *Roseograndinia*, and *H. suiae* is described as a new species. Beyond resolving the taxonomy of *Hyphodermella* itself, this study further clarified that simple phylogenies cannot always accurately place species in appropriate genera. This is an obvious but sometimes omitted phylogenetic practice in recent years (Guan et al. 2020; Zong et al. 2021; Li et al. 2022a; Liu et al. 2022c). We suggest that all fungal taxonomists especially beginners should keep in mind to sample as many comprehensive taxa as possible in phylogenetic, and for that matter morphological analyses (Hawksworth 2020).

## Supplementary Information

**Additional file 1: Alignment S1**. The concatenated alignment of ITS and nLSU.

**Additional file 2: Alignment S2**. The concatenated alignment of ITS, nLSU, *rpb1*, *rpb2* and *tef1α* regions.

## Data Availability

All sequence data generated for this study can be accessed via GenBank: https://www.ncbi.nlm.nih.gov/genbank/.

## Abbreviations

BI: Bayesian inference
BPP: Bayesian posterior probability
BS: Bootstrap
CTAB: Cetyl-trimethyl-ammonium bromide
ML: Maximum likelihood
PCR: Polymerase chain reaction
